# Gene discovery from microbial gene libraries I: protection against reactive oxygen species-driven DNA damage

**DOI:** 10.1128/spectrum.00365-24

**Published:** 2024-09-16

**Authors:** John C. Taylor, Carmen Gu Liu, James D. Chang, Brianna E. Thompson, Anthony W. Maresso

**Affiliations:** 1 Department of Molecular Virology and Microbiology, Baylor College of Medicine, Houston, Texas, USA; 2 TAILΦR: Tailored Antibacterials and Innovative Laboratories for phage (Φ) Research, Baylor College of Medicine, Houston, Texas, USA; 3 Department of Biosciences, Rice University, Houston, Texas, USA; Center of Innovative and Applied Bioprocessing, Mohali, Punjab, India

**Keywords:** DNA damage, DNA stability, DNA library, ROS defense

## Abstract

Reactive oxygen species (ROS) pose a lethal risk for all life forms by causing damage to cell processes, genome-wide DNA damage-driving mutation, replicative instability, and death. Thus, the development of mechanisms to resist or repair ROS-induced DNA damage is critical for the reliable replication of nucleic acids. DNA repair and protection mechanisms have been discovered in all forms of life. However, the vast array of microbes that may harbor novel repair or protection mechanisms, especially bacterial viruses, have not been adequately assessed. Here, we screened a microbial gene library composed primarily of phage open reading frames (ORFs) to uncover elements that overcome a DNA damage blockade. We report the discovery of one such protein, termed F21, which promotes bacterial survival by possibly repairing or protecting DNA in the face of ROS-induced DNA damage.

**IMPORTANCE:**

Discovery of proteins that promote DNA damage repair and protection in the face of reactive oxygen species (ROS) is of vital importance. Our group is in possession of a unique microbial DNA library with which we can screen for undiscovered genes that encode novel proteins with DNA damage repair and protective functions. This library is composed of diverse DNA from a variety of sources, namely bacteriophages, which must be assessed for their novel functions. This work focuses on the discovery of DNA damage repair and protection, but the possibilities for discovery are endless, thus highlighting the significance of this work.

## INTRODUCTION

Reactive oxygen species (ROS) are lethal oxidative species derived from molecular oxygen, including hydrogen peroxide (H_2_O_2_) (1, 2). High levels of ROS induce breaks in DNA and nucleotide alterations, and the accumulation of DNA damage and DNA mutations can eventually lead to reduced replicative stability and integrity, as well as death (3). Thus, ROS present a challenge for DNA replication, a vital cornerstone for all living organisms and viruses. DNA damage repair has been well documented in prokaryotic and eukaryotic organisms and viruses, the most common of which is homologous recombination (HR) (4). One such example in prokaryotes is the RecABCD DNA repair mechanism, wherein RecBCD facilitates degradation of a region of DNA that has been damaged, and RecA creates a nucleoprotein filament that promotes HR (5). In addition, mechanisms to prevent DNA damage from occurring have evolved, such as ROS scavengers that stop ROS from damaging DNA, as well as DNA damage checkpoints (6). Notably, mechanisms of DNA damage repair have also been described in bacteriophages, as in the case of T4 ligase DNA repair (7, 8). Previously, our group assembled microbial DNA libraries, known as Inducible Multi MetagenФmic RecombinanT Libraries (IMMФRTL), which were then introduced into an *Escherichia coli recA*-deficient mutant (∆*recA*), which is susceptible to death by DNA damage without the ability to repair or prevent DNA damage (9). Here, we report the successful isolation and identification of a novel protein associated with repair of ROS-induced DNA damage.

## RESULTS

**Fig 1:**
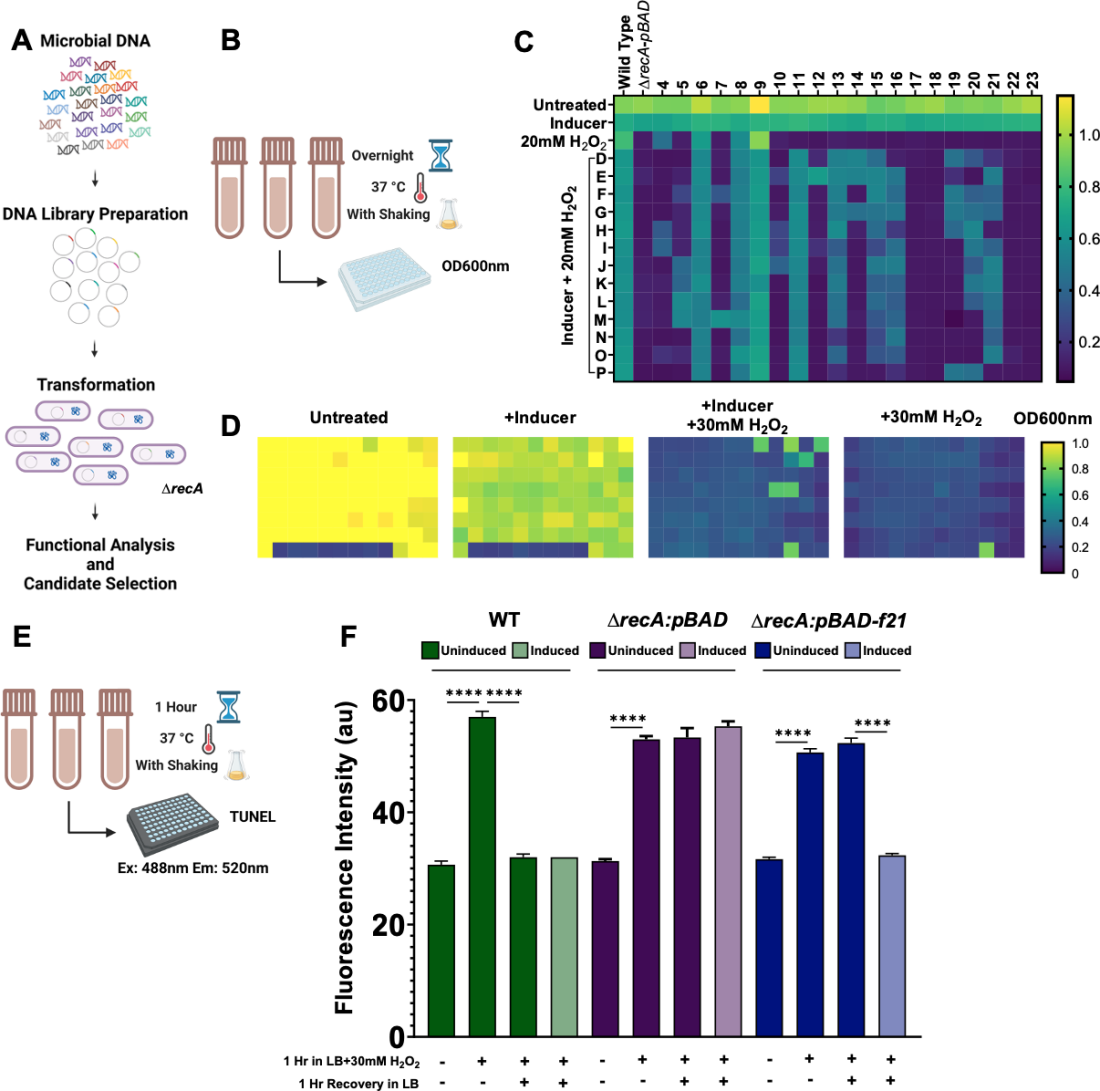
Survival screening for ROS-induced DNA damage repair genes and TUNEL analysis. Schematic of DNA library preparation and transformation of ∆*recA* for functional analysis and candidate selection (**A**). Schematic of survival screening (**B**). Transformants were grown in LB supplemented with 1% arabinose and challenged with 20 mM H_2_O_2_ overnight at 37°C with shaking (**C**). OD600nm was detected the following day to determine death/survival. Hits from the previous screen were grown in LB supplemented with 1% arabinose and challenged with 30 mM H_2_O_2_ overnight at 37°C with shaking, and OD600nm was detected the following day to determine death/survival (**D**). Schematic of TUNEL analysis (**E**). WT, ∆*recA:pBAD*, or ∆*recA:pBAD-F21* bacteria were challenged with or without 30 mM H_2_O_2_ for 1 hour and were allowed to recover and repair DNA damage with or without gene induction with 1% arabinose for 0 or 1 hour while DNA damage was being induced by 30 mM H_2_O_2_ (**F**).

IMMФRTL contains genes from freshwater, seawater, and wastewater biomes. The open reading frames (ORFs) were inserted into the *pBAD* vector expression system, which were then introduced into an *E. coli recA*-deficient mutant (∆*recA*) for functional analysis and candidate selection (Fig. 1A). To discover genes within our library that are implicated in a pro-survival phenotype in the wake of ROS-induced DNA damage, we implemented a high-throughput assay. IMMФRTL-transformed ∆*recA* was grown in the presence of H_2_O_2_ overnight at a dose that is lethal to ∆*recA* but not to wild-type (WT) bacteria, and transformant gene expression was induced with 1% arabinose (Fig. 1B). The preliminary survival assay yielded 85 hits that survived an overnight challenge of 20 mM H_2_O_2_ (Fig. 1C). These hits were then validated in a secondary survival assay in which each hit was challenged overnight with a higher dose of 30 mM H_2_O_2_ to narrow the number of hits for further analysis (Fig. 1D). Validated hits were required to exceed a threshold of OD600nm = 0.4 to be classified as a hit. This secondary validation screen yielded seven hits, and each of the IMMФRTL inserts of the seven validated hits was sequenced to determine their identities. Of these, one sequence presented as the most interesting, which we are calling F21 for short. We selected F21 due to high sequence coverage (99% coverage and 99% identity to an *Alcanivorax recQ* helicase in the IMMФRTL seawater library). This gene is 1,911 base pairs in length, which yields a protein that is 636 amino acids in length. However, during the cloning process, it appears that 552 base pairs of this gene were cloned into *pBAD* in the reverse orientation. This backward sequence, along with the encoded *pBAD* start site, yielded a new gene encoding a 183-amino-acid-long protein (Table 1). We assessed the sequence for possible transcription occurring in the opposite direction and found no viable start site by which the traditional *recQ* gene could be expressed. We then performed BLASTp (NCBI) analysis of the novel F21 amino acid sequence, which revealed no predicted similarity to known proteins. We applied an approach through ESMFold (Meta) (10) to predict the structure of F21 from its amino acid sequence and predict structural homology to other proteins through the DALI structural analysis web server (11). This analysis revealed some structural similarities to a number of proteins, albeit with no higher than 16% identity for any given structure. Given the novelty of this protein, we selected F21 for further analysis.

**TABLE 1:** DNA damage repair hits: sequences and similarity prediction

Protein	Amino acid sequence^*a*^	Length (aa)	Predicted similarity to known proteins^*b*^
F21	MTMTSSPFHDSSRITYLLQPLDVALLTNTKLLCDLCRQRLRISWTIDHPIALAHPCHTHLNHALLLKIANRLENSHPIDLQLVRHGNRFQSEPDAPLFTRSKDLPQKIRSLMVELSQHLSLRYLKCQLVLRIERRCRTNNPVQLQIGQQGINNQRADIKLPRQFMHRKVELRRLIHQPANEPG	183	None
RecQ	MLTLQQHLQQTFKLQGFRPGQERVINALLAGRSALAVFPTGGGKSLCYQLPALMLDGVTLVVSPLIALMKDQVDQLHRLGIAAARLDSSVDAEQLQSIYRGLEDGSIKLLYVAPERLANERFLARLKRLSISMMAVDEAHCVSEWGHNFRPDYLKLAQLAKELEVGRVLALTATATPQVAQQICDSFAIAPADHVQTGFFRPNLALSVRPYNVEERGRALLEALQARPSEPAIVYVTLQKTATQVAALLSEQGLNAQAYHAGLKGEERHRVQEAFMSGQVNIVVATIAFGMGIDKADIRGIYHYNLPKSLENYMQEIGRAGRDGEPSQCVLLASSDDLTMLENFSYGDTPDSEALAGLIGWLMNQPAQFDLAVHELSGQFDVRPLVINTLLTYLELDGIIRSTAPFYTEYKLAFQISQAEVLAQFDHERADFLRQVFAAGKEGRIWLTLKPIAVADQLQVDRVRILKAIGYLEQQGMVEVRVAGVRQGYRMVNRPADPQALTAQIAEQFRVREQRDIQRLQQVCNWAQSSDCYQRGLVGYFGEQLPQPCGQCSACKGEHQRFPERHRTAPDSAVILAVIAQRHGALATPRQLARFLCGISSPRASRARLARHPSFGSQMEVPFAEVLATCRAMLEP	636	ATP-dependent DNA helicase RecQ (Accession: WP_046961840.1)

^*a*^ Nucleotide sequences were obtained through Sanger sequencing by GeneWiz (Azenta) and were translated via a nucleotide translation tool (Expasy).

^*b*^ Amino acid sequences were subjected to BLASTp (NCBI) to determine predicted similarity to known proteins.

We sought to analyze the role of F21 as it pertains to the prevention and repair of ROS-induced DNA damage. To accomplish this, we implemented a TUNEL (12) assay in which WT, ∆*recA:pBAD*, or ∆*recA:pBAD-F21* bacteria were challenged with or without 30 mM H_2_O_2_ for 1 hour and were allowed to recover with or without gene induction with 1% arabinose for 0 or 1 hour (Fig. 1E). As expected, all bacteria that were not allowed time to recover exhibited a significant increase in DNA damage compared to bacteria grown in the absence of 30 mM H_2_O_2_ (Fig. 1F; ****P < 0.0001). When allowed 1 hour for recovery, the WT strain exhibited no increase in DNA damage regardless of induction, and the mutant retained its increase in DNA damage regardless of induction compared to its untreated counterparts, as expected. However, when given the opportunity to recover with gene induction, DNA damage in ∆*recA:pBAD-F21* was significantly reduced compared to its uninduced counterpart (****P < 0.0001), indicating that F21 plays a crucial role in either preventing or repairing DNA damage induced by ROS. We next sought to determine if this phenotype was exclusive to extracellularly added ROS by repeating the TUNEL assay in *pBAD-F21*-transformed Hpx^-^ *E. coli* mutants, which lack multiple catalases (13) (Fig. S1A). This mutant carries an endogenous level of DNA damage, and F21 expression significantly reduced this (Fig. S1B; ****P < 0.0001). Taken together, these results indicate that F21 is involved in the bypass or repair of ROS-induced DNA damage to promote bacterial survival.

**Fig 2:**
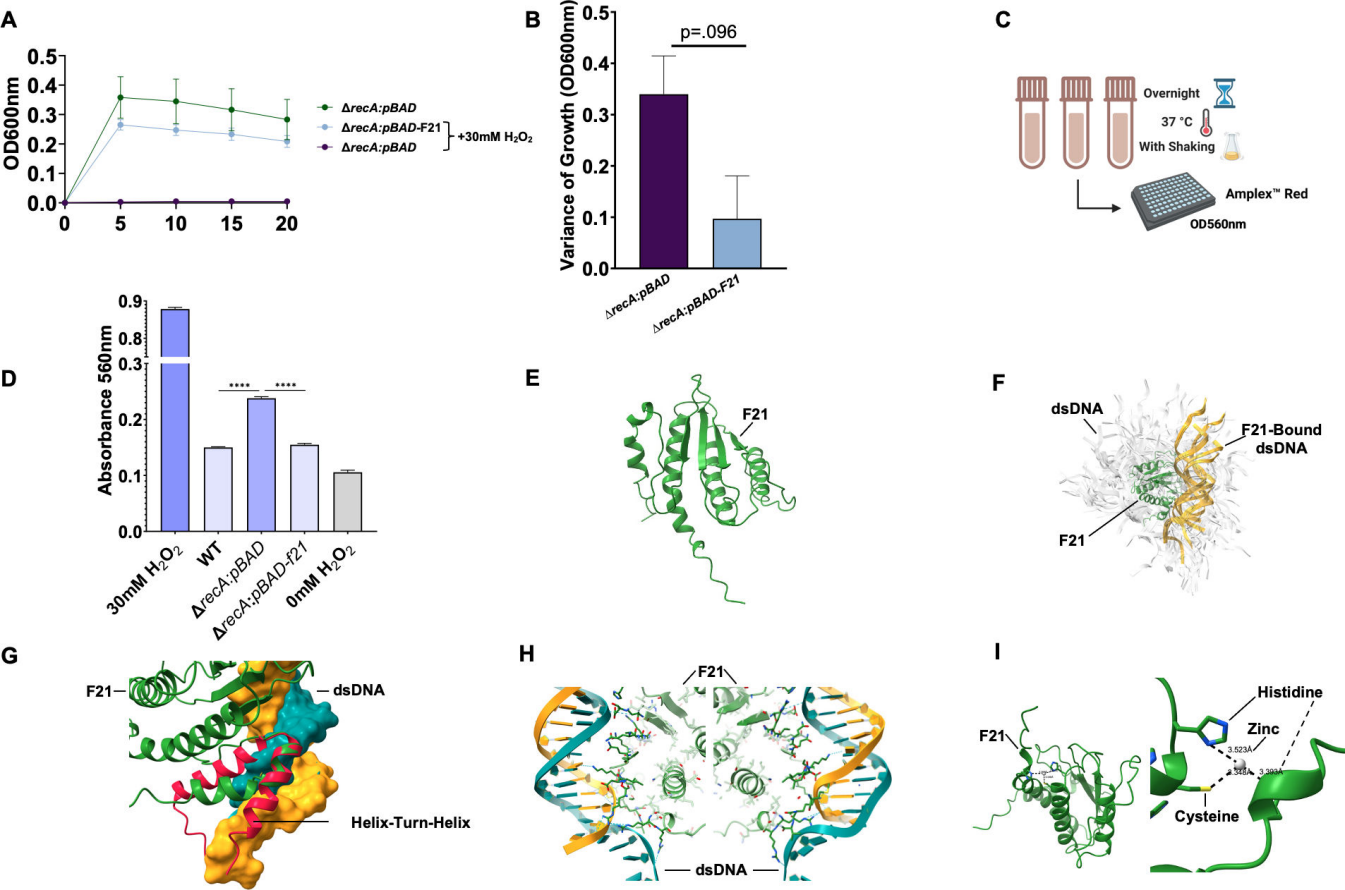
F21 rescue of ROS-induced growth inhibition, reduction of H_2_O_2_ in culture, and structural analysis of F21. Twenty-hour growth curve of ∆*recA:pBAD-F21* and ∆*recA:pBAD* bacteria in the presence and absence of 30 mM H_2_O_2_; expression induced with 1% arabinose (**A**). Variance in bacterial growth for F21 and mutant bacteria was calculated as follows: variance of growth OD600nm = OD600nm_Max Untreated_ – OD600nm_Max Treated_ (**B**). Schematic for H_2_O_2_ detection following overnight growth (**C**). H_2_O_2_ levels with an Amplex Red H_2_O_2_ detection kit following overnight growth of WT, mutant, and F21 bacteria in 30 mM H_2_O_2_ with 1% arabinose for gene expression were assessed (**D**). Ribbon diagram of F21’s predicted structure (**E**). Top 100 binding modes between F21 (green) and dsDNA (grey), with top five binding modes highlighted (yellow) (**F**). Alignment of F21 (green) and solved helix-turn-helix motif (red) (**G**). F21 residues that interact within 5 Å of DNA (**H**). Cysteine/histidine coordinating zinc pocket of F21 (**I**).

We sought to understand F21’s effects on the pro-survival phenotype during H_2_O_2_ challenge in the presence or absence of 30 mM H_2_O_2_. As expected, ∆*recA:pBAD-F21* grew in the presence of 30 mM H_2_O_2_, whereas 30 mM H_2_O_2_ treatment disallowed the growth of the mutant (Fig. 2A). We calculated the maximum variance of growth exhibited by both ∆*recA:pBAD-F21* and ∆*recA:pBAD* when challenged with 30 mM H_2_O_2_ compared to untreated ∆*recA:pBAD* (Fig. 2B and defined in the Methods). Unsurprisingly, H_2_O_2_ challenge decimated mutant growth, resulting in a high variance of growth between challenged and unchallenged mutant growth. However, this variance was decreased in ∆*recA:pBAD-F21* (Fig. 2B; *P* = 0.096). Thus, one may surmise that the effect of F21 gene expression yields a pro-survival phenotype due to its protection of DNA integrity. Accordingly, we assessed H_2_O_2_ levels by way of an Amplex Red peroxide detection assay (Thermo Fisher) following overnight growth of WT, mutant, and F21 bacteria in 30 mM H_2_O_2_ (Fig. 2C). Expectedly, the WT bacteria were capable of significantly decreasing H_2_O_2_ in culture compared to the mutant (Fig. 2D; ****P < 0.0001). Importantly, this same reduction of H_2_O_2_ in culture was observed in ∆*recA:pBAD-F21* (*P* < 0.0001). These results indicate that F21 gene expression promotes tolerance to H_2_O_2_ challenge, allowing for the bacteria to reduce H_2_O_2_ in solution overnight. It is not known at this point if the reduction in peroxide drives DNA repair or if the reduction in damaged DNA better helps the cells to cope with excess peroxide.

To further expand upon our knowledge of F21 and understand its association with reduced DNA damage, we implemented a bioinformatics approach to assess the structure of F21 to predict its function. Predicted structure is dominated by anti-parallel beta-sheet that forms into a stable platform that aids consistent target binding (Fig. 2E). We examined F21’s potential for DNA binding using a pyDockDNA (14) web server to randomly generate binding modes between F21 and linear dsDNA (PDB: 4YNQ) and score them using physical parameters (Fig. 2F). Higher-ranked binding modes all interacted to a region that closely resembled a helix-turn-helix DNA motif (PDB: 7T5W) (15) that showed high structural similarity upon alignment for residues 157–169 (RMSD: 1.37 Ǻ; TM-score: 0.44) (Fig. 2G). We also used a DRNAPred algorithm (16) that relies on amino acid sequences to calculate the probability of DNA interaction by residue. DRNAPred assigned probabilities of DNA interaction for residues with a high DNA interaction probability for residues 162–168 (average probability of 0.73). These binding modes all had hydrogen bonds between dsDNA and a region between the beta-sheet and C-terminal end, with the highest scoring solution with seven potential hydrogen bonds (Fig. 2H). Given that many DNA binding molecules use metal ions such as zinc as essential DNA binding co-factors (17), we searched for such a metal binding pocket on F21 and found that F21 does indeed have a predicted metal binding pocket in which zinc coordinates canonical binding sidechains within roughly 3.5 Å of each residue (His9, His54, and Cys56; Fig. 2I). Through this analysis, we were able to predict the binding of F21 to DNA and identify the residues and structural characteristics harbored by F21 to accomplish what we propose is a protective and reparative function toward damaged DNA. To test the hypothesis that F21 binds DNA, we performed a DNA-binding assay in which MluCI-digested *E. coli* genomic DNA was incubated with F21-expressing bacterial lysate. The bound DNA was then run on a DNA agarose gel to observe any band shift indicative of F21 binding to genomic DNA. DNA from only the recA mutant, with or without the expression vector or induction, resulted in a diffuse smear at higher molecule sizes after EtBr staining (lanes 1–3, Fig S1C). The inclusion of *recA* mutant DNA with the expression vector containing the F21 insert yielded the same observation. However, when this construct was induced and F21-expressing bacterial lysate was added to the DNA, a significant band shift in DNA was observed, which was absent from all control groups (arrows).

## DISCUSSION

We utilized a microbial gene library (IMMФRTL) in a *recA*-deficient strain to identify survivors in the presence of oxidative DNA stress, which successfully yielded an exciting new gene of interest, F21. We then implemented functional and computational analyses to discover that F21 significantly repairs and protects DNA from ROS-induced DNA damage and rescues growth, all the while contributing to decreased ROS in culture, further benefiting growth. Moreover, a homology search and structure prediction led us to the understanding that F21 is a unique and novel protein that is predicted to bind DNA.

Notably, F21 was discovered by screening of ∆*recA*. Thus, when considering the mechanism by which F21 is aiding in the facilitation of DNA damage prevention and repair, we must consider that F21 is likely acting in a way that compensates for the loss of *recA*. Importantly, F21 does not bear significant homology to the RecA protein, and it is thus unlikely that F21 is directly filling the role of RecA. Rather, it is more likely that F21 is acting in a unique capacity to compensate for the loss of RecA. We speculate that F21 binds to DNA and protects it, in a mechanism to be determined, from further oxidative stress caused by H_2_O_2_. Given that *E. coli* bacteria possess their own enzymes that break down ROS (18), it is possible that F21 aids in protecting DNA from oxidative stress caused by H_2_O_2_ and, thus, ensures DNA integrity so that endogenous catalases can break down peroxide. This would explain the protective function of F21 in mitigating DNA damage by H_2_O_2_ (Fig. 1F), as well as the rescued bacterial growth and reduced peroxide in solution following overnight growth (Fig. 2A and B). This theory is further supported by supplemental evidence indicating that both WT and *∆recA* are capable of surviving a sublethal dose of 10 mM H_2_O_2_ (Fig. S2A). Additionally, both WT and *∆recA* are capable of reducing H_2_O_2_ in overnight culture when challenged with 10 mM H_2_O_2_ (Fig. S2B). This indicates that *∆recA* still retains its endogenous catalase activity and propensity for survival when the dose of H_2_O_2_ is sublethal. Moreover, it appears that F21 expression significantly reduced DNA damage in Hpx^-^ *E. coli*, lacking several catalases (S1B), indicating the role of F21 to protect DNA from ROS damage. Because H_2_O_2_ is the substrate of catalases (19), this further accentuates the role of F21 to protect DNA from ROS, namely H_2_O_2_. However, the mechanism by which DNA is repaired in association with the induction of F21 expression requires further investigation. Given that F21 is a novel product that displays no apparent similarities to anything in the public database, as indicated by BLASTp and DALI analysis, we cannot rule out that it is not directly repairing DNA. However, both bioinformatics and our DNA-binding experiment (Fig. S1C) support the role for F21 to bind DNA. This, combined with reduced DNA damage during F21 expression and reduced H_2_O_2_ following overnight culturing, tells an intriguing story of a protein that appears to bind and protect DNA, thus enhancing F21-expressing bacterial survival through reduced DNA damage. This work creates an important basis for which further research may be accomplished, such as investigating other mechanisms of DNA damage, including the investigation of other ROS as well as other DNA damaging agents like genotoxins and UV light. Thus, its full characterization is required to determine its exact role in the phenomenon of maintaining DNA integrity, hence the submission of this paper under the category of a novel observation.

## MATERIALS AND METHODS

### Bacterial storage, growth conditions, and handling

Bacteria were stored, grown, and handled as previously described (20). Briefly, *recA*-deficient and WT *E. coli* were obtained from the KEIO collection of the National BioResource Project (21, 22) and were electroporated with IMMФRTL. Bacterial stocks were prepared in 25% glycerol and requisite antibiotics for plasmid retention. Bacteria were grown in lysogeny broth (LB), supplemented with requisite antibiotics for plasmid retention, 1% arabinose for the induction of plasmid insert expression, or H_2_O_2_ to provide DNA damage where applicable.

#### Survival screening

Overnight bacterial cultures were centrifuged (5,000 × *g* for 10 minutes) and adjusted to final concentrations of OD600nm = 0.35 in the presence of 1% arabinose or H_2_O_2_. Cultures were then incubated overnight at 37°C with shaking at 220 rpm. The following day, OD600nm was assessed to determine survival. In the preliminary screen (Fig. 1C), mixed-population cultures were pipetted into wells in respective columns and were diluted row-by-row to obtain individual transformants for further analysis. Each column (4–23) represents different mixed-population IMMФRTL cultures, and subsequent rows (D–P) represent the dilutions of those cultures from which individual transformants can be isolated and assessed following screening. Notably, columns in which bacterial growth was observed in the presence of H_2_O_2_ without the inducer (columns 4, 6, 8, and 9) were not assessed. Hit wells were only assessed under the following parameters: (i) growth was observed in LB alone and in the presence of the inducer; (ii) growth was not observed in the presence of H_2_O_2_ without the inducer.

#### Growth kinetics

Bacterial growth (20 hours) was assessed with readings at *t* (hours) = 0, 5, 10, 15, and 20. Variance in growth was calculated as follows: variance of growth OD600nm = OD600nm_10 Hours Untreated_ – OD600nm_10 Hours Treated_.

### Functional screening

#### TUNEL

TUNEL analysis was performed as described previously (12). Briefly, overnight cultures were diluted to OD600nm = 0.35, challenged with H_2_O_2_ for 1 hour at 37°C with shaking at 220 rpm, followed by a recovery period of 1 hour at 37°C with shaking at 220 rpm, with or without gene induction with 1% arabinose. Bacteria were collected, centrifuged at 5,000 × *g* for 10 minutes, followed by treatment with a TUNEL assay kit (Abcam, cat# ab66108). Fluorescence intensity was observed at Ex/Em = 488 nm/520 nm.

#### Amplex Red

Amplex Red analysis was performed as described previously. Briefly, overnight cultures were diluted to OD600nm = 0.35, challenged with 30 mM H_2_O_2_, and induced with 1% arabinose overnight at 37°C with shaking. Supernatant was collected the next day following centrifugation at 5,000 × *g* for 10 minutes. Supernatants were then treated with the Amplex Red Hydrogen Peroxide/Peroxidase Assay kit (Thermo Fisher; cat# a22188). Fluorescence intensity was measured at Ex/Em = 530 nm/590 nm.

### Plasmid insert sequencing

Candidates were grown overnight, with requisite antibiotics, at 37°C with shaking at 220 rpm. The following day, the bacterial pellets were collected via centrifugation at 5,000 × *g* for 10 minutes, and plasmids were collected via a plasmid purification kit (Omega Bio-Tek; cat# d6943-01). Collected plasmids were then submitted for Sanger sequencing by Genewiz (Azenta). Insert DNA sequences were then matched to IMMФRTL. BLASTp (NCBI) was used to predict similarity to known proteins in the NCBI database.

### Protein structure prediction and DNA binding analysis

ESMFold (Meta) (10) was used to predict protein structure, DALI (11) was used for structural homology search, and UCSF ChimeraX (23) was used to visualize each structure, as well as surface charge and hydrophobicity. F21 binding to tetraplex DNA was modeled using PyDockDNA (14) and was visualized with UCSF ChimeraX (23).

#### Electrophoretic mobility shift assay

Purified *E. coli* genomic DNA was digested with MluCI as described in the manufacturer’s protocol (New England Biolabs; cat#-R0538L). Bacterial lysates were prepared by vigorously vortexing bacteria with glass beads for 5 minutes, followed by centrifugation at max speed for 5 minutes. Resulting lysate was mixed with genomic DNA and incubated with Component E of EMSA Kit per the manufacturer’s protocol.

### Statistics and data analysis

Survival screens were performed once for the purpose of candidate selection, followed by a secondary survival screen at a higher ROS concentration for result validation. Subsequent experiments for validation and phenotype analysis were performed using three biological replicates. Where applicable, graphs were generated, and unpaired two-tailed *t*-tests were used to determine statistical significance in Prism 9 (GraphPad). *P < 0.05, **P < 0.01, ***P < 0.001, ****P < 0.0001. Our final summary model and all schematics were generated using BioRender.

## SUPPLEMENTAL MATERIAL

Fig. S1 spectrum.00365-24-s0001.tiff Schematic of Hpx-TUNEL analysis (A). Transformants were treated with or without 1% arabinose to induce gene expression for 1 hour (B). MluCI-digested genomic DNA from ∆*recA* was incubated with lysate from F21-expressing or non-expressing bacteria and ran on a DNA gel to observe band shifts indicative of DNA binding (C).

Fig. S2 spectrum.00365-24-s0002.tiff Survival analysis of WT and ∆*recA* overnight cultures treated with a sublethal dose of 10 mM H_2_O_2_ compared to untreated controls (A). Amplex Red assay of WT and ∆*recA* overnight cultures in LB + 10 mM H_2_O_2_ (B).

ASM does not own the copyrights to Supplemental Material that may be linked to, or accessed through, an article. The authors have granted ASM a non-exclusive, world-wide license to publish the Supplemental Material files. Please contact the corresponding author directly for reuse.
