# Supplementary figures and images for: Gene discovery from microbial gene libraries I: protection against reactive oxygen species-driven DNA damage
Source: Microbiol Spectr. 2024 Sep 16;12(11):e00365-24. doi: 10.1128/spectrum.00365-24 (PMC11536983; doi:10.1128/spectrum.00365-24)

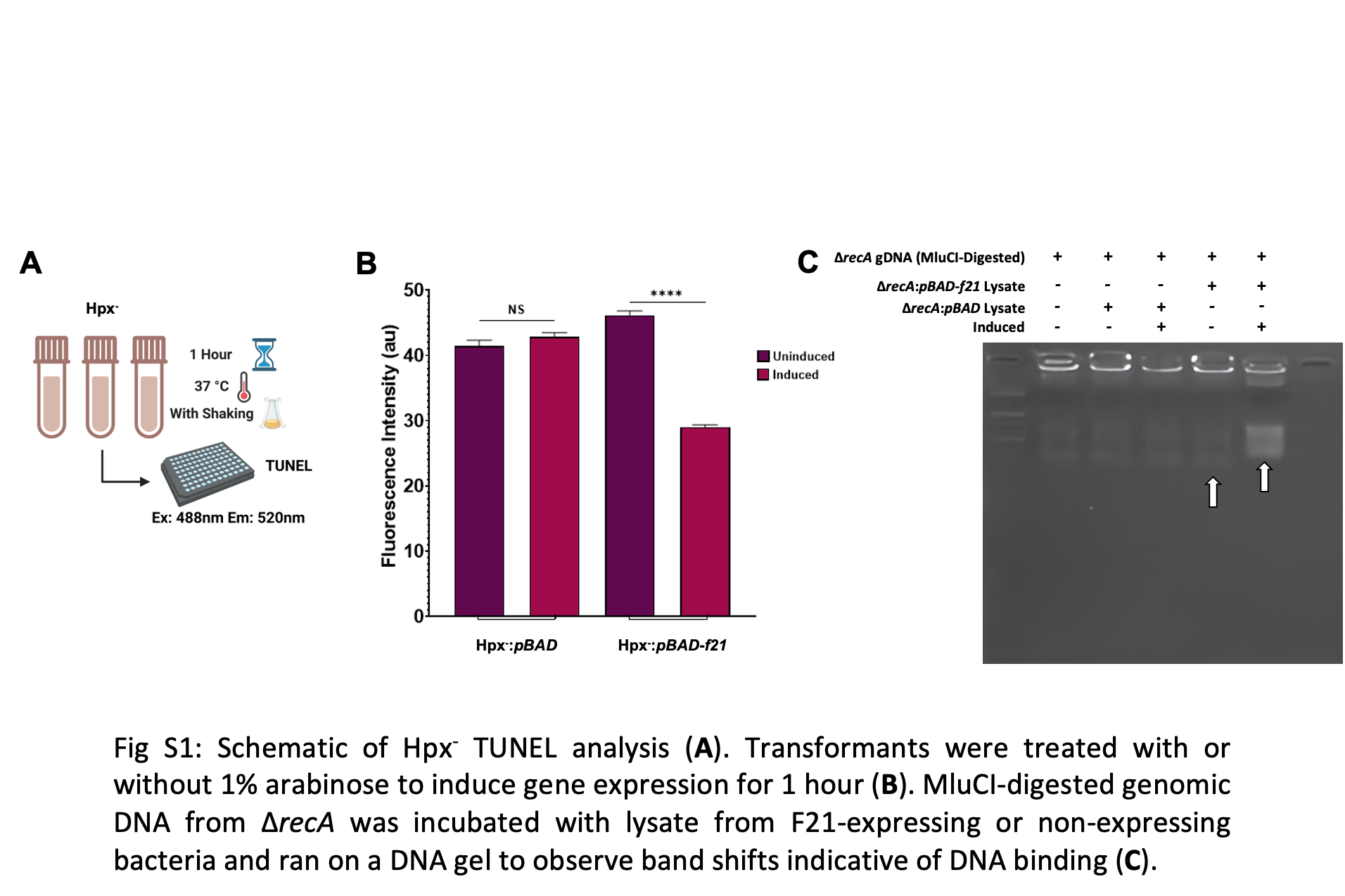

Supplement: Fig. S1 — Schematic of Hpx-TUNEL analysis (A). Transformants were treated with or without 1% arabinose to induce gene expression for 1 hour (B). MluCI-digested genomic DNA from ∆recA was incubated with lysate from F21-expressing or non-expressing bacteria and ran on a DNA gel to observe band shifts indicative of DNA binding (C). [file spectrum.00365-24-s0001.tiff]

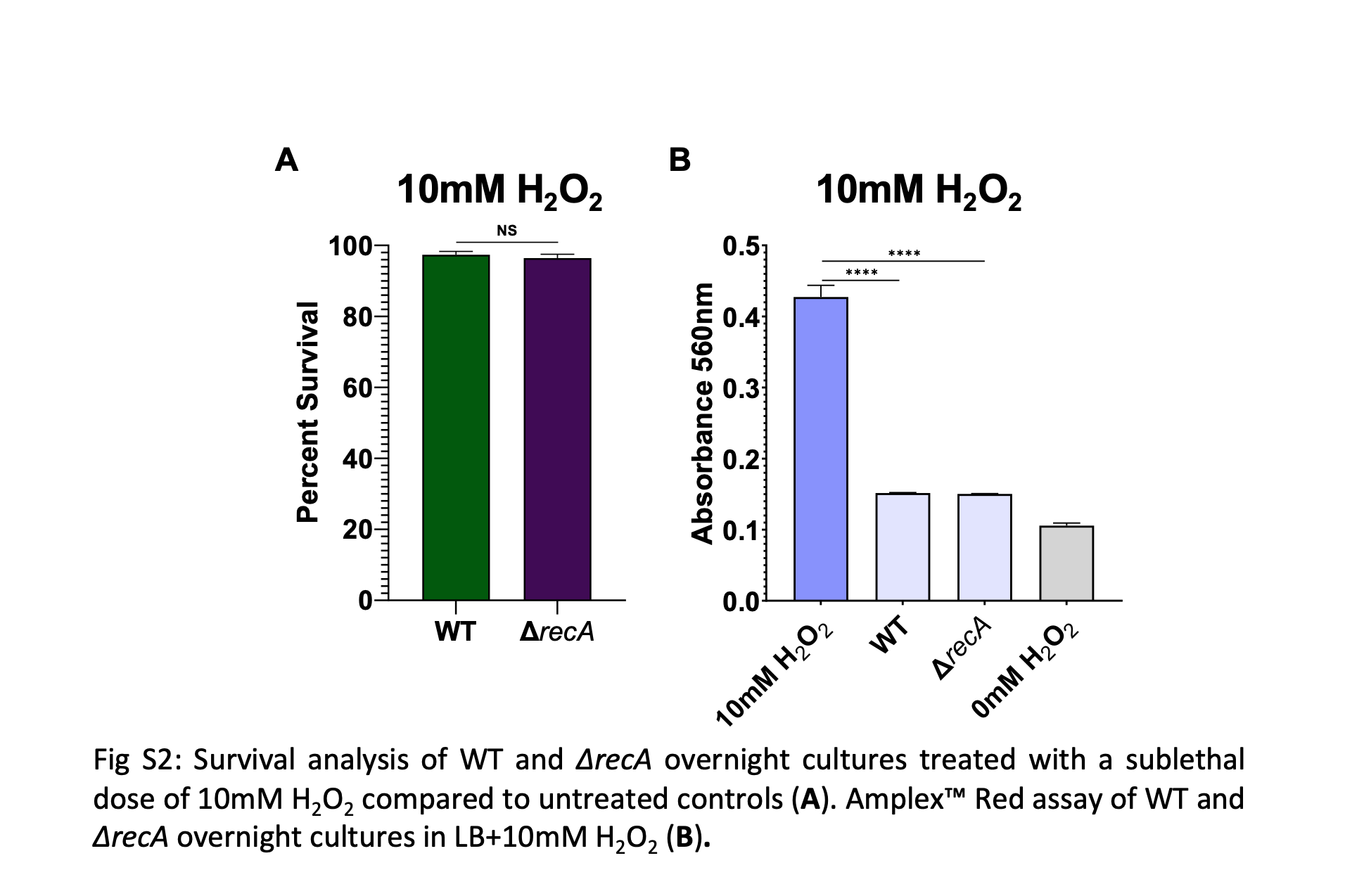

Supplement: Fig. S2 — Survival analysis of WT and ∆recA overnight cultures treated with a sublethal dose of 10 mM H2O2 compared to untreated controls (A). Amplex Red assay of WT and ∆recA overnight cultures in LB + 10 mM H2O2 (B). [file spectrum.00365-24-s0002.tiff]
